# Urinary 1-hydroxypyrene and malondialdehyde in male workers in Chinese restaurants

**DOI:** 10.1136/oem.2007.036970

**Published:** 2008-10-13

**Authors:** C-H Pan, C-C Chan, Y-L Huang, K-Y Wu

**Affiliations:** 1 Institute of Occupational Medicine and Industrial Hygiene, College of Public Health, National Taiwan University, Taipei, Taiwan; 2 Department of Biomedical Laboratory Science, Kaohsiung Medical University, Kaohsiung, Taiwan; 3 Division of Environmental Health and Occupational Medicine, National Health Research Institute, Miaoli, Taiwan

## Abstract

**Objectives:**

To assess internal dose and oxidative stress in male restaurant workers exposed to polycyclic aromatic hydrocarbons (PAHs) from cooking oil fumes (COFs) in Chinese restaurants.

**Methods:**

The study participants included 288 male restaurant workers (171 kitchen and 117 service staff) in Chinese restaurants in Taiwan. Airborne particulate PAHs were measured over 12 h on each of two consecutive work days and then identified using high performance liquid chromatography. Urinary 1-hydroxypyrene (1-OHP) measurements were used to indicate COF exposure, and urinary malondialdehyde (MDA) was adopted as an oxidative stress marker. Multiple regression models were used to assess the relationship between MDA and 1-OHP levels after adjusting for key personal covariates.

**Results:**

Summed particulate PAH levels in kitchens (median 23.9 ng/m^3^) were significantly higher than those in dining areas (median 4.9 ng/m^3^). For non-smoking kitchen staff, mean MDA and 1-OHP levels were 344.2 (SD 243.7) and 6.0 (SD 8.0) μmol/mol creatinine, respectively. These levels were significantly higher than those for non-smoking service staff, which were 244.2 (SD 164.4) and 2.4 (SD 4.3) μmol/mol creatinine, respectively. Urinary 1-OHP levels were significantly associated with work in kitchens (p<0.05). Furthermore, urinary MDA levels were significantly associated with urinary 1-OHP levels (p<0.001) and working hours per day (p<0.05).

**Conclusions:**

These findings indicate that urinary 1-OHP and MDA levels reflect occupational exposure to PAHs from COFs and oxidative stress in workers in Chinese restaurants.

Cooking oil fumes (COFs) are created and released into the environment when food is fried, stir fried or grilled using cooking oil at high temperatures. The degradation of sugar and fat, and the pyrolysis of proteins and amino acids during high temperature treatment of food can produce harmful degraded materials^1^ such as polycyclic aromatic hydrocarbons (PAHs),^2^ aromatic amines,^3^ nitro-polycyclic aromatic hydrocarbons,^4^ benzene^5^ and aldehydes.^6^ ^7^ Traditional Chinese cooking makes frequent use of stir frying and deep frying which generate significant amounts of COFs.^8^

Because the adverse health effects of COFs are related to long-term exposure, a biological monitoring approach assessing the internal dose of an individual is the preferred approach for examining occupational exposure to COFs. One indicator of COF exposure for restaurant workers is the presence of metabolites of PAHs, such as 1-hydroxypyrene (1-OHP). Studies have identified urinary 1-OHP as a good biological marker of PAH exposure in fire-fighters,^9^ iron foundry workers^10^ and coke oven workers.^11^ ^12^ This study investigates whether urinary 1-OHP and work in kitchens are good indicators of COF exposure in workers in Chinese restaurants.

Increased induction of lipid peroxidation of lung epithelial cells and oxidative stress following exposure to COFs have been reported in an in vitro^8^ and a cross-sectional study,^13^ respectively. Notably, two studies conducted in Western countries identified an increased risk of lung cancer in male restaurant cooks, even after adjusting for tobacco use.^14^ ^15^ Oxidative stress is defined as a disequilibrium between pro-oxidant and anti-oxidant systems in intact cells resulting from oxygen-using metabolic reactions; the pro-oxidant and anti-oxidant systems continuously generate and detoxify oxidants during normal aerobic metabolism. Lipid peroxidation is a type of oxidative stress and can be defined as the oxidative deterioration of lipids containing carbon–carbon double bonds. Plasma malondialdehyde (MDA) is a biological marker of lipid peroxidation resulting from oxidative stress.^16^ Previous studies have demonstrated relationships between lipid peroxidation and atherosclerosis,^17^ aging,^18^ rheumatoid arthritis,^19^ diabetes mellitus^20^ and cancer.^21^ Studies also demonstrated that MDA, a stable aldehydic product of lipid peroxidation contained in biological samples such as urine, hair or blood, can reflect global oxidative status in the human body.^22^ ^23^ This study assesses internal dose using urinary 1-OHP and oxidative stress using urinary MDA in male restaurant workers exposed to PAHs from COFs in Chinese restaurants.

## METHODS

### Study subjects

In 2005, 372 male restaurant workers employed for at least 1 year in 19 Chinese restaurants in Taiwan were contacted via two trade unions. A total of 288 restaurant workers who completed both a questionnaire survey and a health check-up were then recruited (response rate 77%). The 85 non-participating workers could not attend pre-arranged health check-ups owing to work commitments. Trained interviewers met the participants between July 2005 and December 2005 and using a questionnaire collected data on age, work experience, main cooking methods used, height, weight, employment in kitchen work versus other restaurant employment, health status and life style (including smoking, cooking at home, second-hand smoke exposure and use of respiratory protection devices). The variables smoking, cooking at home and second-hand smoke exposure were defined as having to be present on at least 4 days each week. Subjects who reported smelling cigarette smoke at any time in any place were counted as having been exposed to second-hand smoke that day.

Based on job titles, the 288 restaurant workers were divided into two groups. Kitchen staff included chefs, sous chefs, sauce chefs, executive chefs and assistant cooks, all of whom were relatively close to the sources of COFs in restaurants. Service staff included reception staff, cashiers, waiters and valets, who had relatively low COF exposure in restaurants. Spot urine samples were collected from all subjects during their 2005 annual physical examination. All participants were asked to wash their hands before urine collection to avoid contamination. The Institute Review Board of the National Health Research Institutes in Taiwan approved this study. All subjects provided informed consent.

### Exposure measurement

Daily area monitoring was conducted for particulate PAHs in kitchens and dining areas over two consecutive days in all 19 Chinese restaurants in this study. Particulate PAHs in the work place were sampled using IOM (Institute of Occupational Medicine, UK) samplers with glass fibre filters (diameter 25 mm, pore size 0.7 μm) at a flow rate of 2.0 l/min. The samplers were placed near the breathing zone of the workers and duplicate samples were obtained for each sampling location. Airborne particulate PAHs were measured for 12 h on each of two consecutive work days, and then analysed by high performance liquid chromatography (HPLC). Five PAH species (pyrene, benzo(k)fluroranhene, benzo(a)pyrene, benzo(ghi)perylene and dibenzo(a,e)pyrene) were quantified via HPLC. Each sample collected was extracted using 2 ml n-hexane in an ultrasonic bath for 20 min and 4 ml 5% NaOH was added before centrifuging at 3000 rpm for 20 min. Subsequently, dimethyl sulfate (DMS) was added to a 1.5-ml suspension solution and condensed via nitrogen gas (N_2_). PAH content in the final solution was determined by a Shimadzu HPLC system (Kyoto, Japan) with a system controller (SCL-10A) and a fluorescent detector (RF-10AXL) equipped with a semi-micro column (Kaseisorb LC ODS-60-5, 4.6 mm×250 mm). The mobile phase consisted of a CH_3_CN/H_2_O (9:1) solution with a flow rate of 1.0 ml/min. The detection limits were determined via seven repeated analyses of the lowest standards for each PAH species. The coefficient variation for these repeated analyses was less than 2% for all five PAHs. The detection limits were 0.28 pg for pyrene, 0.72 pg for benzo(k)fluroranhene, 0.28 pg for benzo(a)pyrene, 0.63 pg for benzo(ghi)perylene and 0.43 pg for dibenzo(a,e)pyrene.

### Urinary 1-OHP and MDA

Urinary 1-OHP was analysed via HPLC using a fluorescent detector.^12^ The detection limit was found to be approximately 0.1 μg/l based on seven repeated analyses of 1-OHP at 15.0 μg/l, and the variation in the coefficients of repeated analyses for urinary 1-OHP was less than 10%. Urinary MDA concentration was measured with an HPLC system (JASCO, Model 980-PU, Tokyo, Japan) using a C_18_ column and ultraviolet-visible detector at 532 nm (JASCO UV-975). The mobile phase comprised methanol/potassium phosphate (9:11) buffer and the flow rate was 1.2 ml/min. The within-run and run-to-run precisions of MDA in urine were assessed. The samples were analysed for MDA based on thiobarbituric acid (TBA) reaction, with HPLC separation of the MDA (TBA)_2_ adduct, using tetraethoxypropane as a standard. A detection limit of 0.06 μg/l was obtained from seven repeated analyses of deionised water and the coefficient variation in repeated analyses was less than 10%. The urinary 1-OHP and MDA levels of each individual were corrected according to urine creatinine values, which were measured using an automated method based on the Jaffe reaction.^24^

### Statistical methods

Student’s t and χ^2^ statistics were used to compare personal covariates, urinary MDA and 1-OHP between kitchen and service staff. Furthermore, non-parametric tests with Mann–Whitney U test were used to compare differences in work place PAH levels between kitchen and service staff. Urinary MDA and 1-OHP concentrations were log-transformed to normalise their distributions prior to regression analysis. All data from 154 non-smoking restaurant workers were then included in multiple linear regression models to identify significant predictors of non-smoking worker urinary MDA and 1-OHP concentrations, respectively. The level of statistical significance was set at α = 0.05 for all tests. All data were analysed using SAS v 9.1.

## RESULTS

The kitchen staff averaged 4.1 (SD 2.3) cooking hours in the work place per day. Table 1 compares personal characteristics, work experience and health behaviour between kitchen and service staff. Deep frying, stir frying, grilling, steaming and stewing were the five main cooking methods used by kitchen staff in the surveyed Chinese restaurants. Steaming and stewing were the two main cooking methods used by both kitchen and service staff when cooking at home. There were no significant differences in height, weight, BMI, smoking, number of cigarettes smoked per day, or exposure to second-hand smoke between the kitchen and service staff. None of the 288 restaurant workers wore respiratory protection devices (eg, masks) during the work period.

**Table 1:** Comparison of personal characteristics, work experience and health behaviour between 288 male restaurant workers

	Service staff (n = 117)	Kitchen staff (n = 171)
Personal characteristics, mean (SD)		
Age (years)	35.6 (12.5)	39.3 (11.0)†
Height (cm)	167.8 (6.3)	167.1 (6.5)
Weight (kg)	69.7 (10.5)	71.2 (11.6)
Body mass index (kg/m^2^)	24.8 (3.5)	25.5 (3.6)
Work experience, mean (SD)		
Working years	12.2 (11.1)	15.0 (9.9)*
Work days per week	5.3 (0.4)	5.4 (0.3)*
Work hours per day	8.5 (1.8)	9.1 (2.5)*
Cooking hours at work per day	0.0 (0.0)	4.1 (2.3)
Main cooking methods at work, n (%)		
Deep frying	0 (0.0%)	97 (56.7%)
Stir frying	0 (0.0%)	120 (70.2%)
Grilling	0 (0.0%)	73 (42.7%)
Steaming	0 (0.0%)	95 (55.36)
Stewing	0 (0.0%)	95 (55.36)
Health behaviour, n (%) or mean (SD)		
Smoking (⩾4 days per week)	56 (47.9%)	78 (45.6%)
Number of cigarettes smoked per day	5.5 (SD 7.4)	6.3 (SD 9.6)
Cooking at home (⩾4 days per week)	8 (6.8%)	62 (36.3%)‡
Second-hand smoke exposure	45 (38.5%)	74 (43.3%)
(⩾4 days per week)		

*Kitchen staff differ significantly from service staff at p<0.05 by Student’s t test; †kitchen staff differ significantly from service staff at p<0.01 by Student’s t test; ‡kitchen staff differ significantly from service staff at p<0.001 by χ^2^ test.

Table 2 lists particulate PAH levels in the kitchens and dining areas of 19 Chinese restaurants. The median levels of pyrene, benzo(k)fluroranhene, benzo(a)pyrene, benzo(ghi)perylene and dibenzo(a,e)pyrene in the kitchens were significantly higher than those in the dining areas. The median levels of summed PAHs in the kitchens were also significantly higher than those in the dining areas.

**Table 2:** Comparisons of particulate polycyclic aromatic hydrocarbon (PAH) levels (median, ng/m^3^) between kitchens and dining areas in 19 Chinese restaurants

PAH	Dining areas(n = 76)	Kitchens(n = 76)
Pyrene	0.2	3.3*
Benzo(k)fluroranhene	0.2	1.4*
Benzo(a)pyrene	1.2	5.9*
Benzo(ghi)perylene	0.6	5.2*
Dibenzo(a,e)pyrene	1.8	8.7*
Summed PAHs†	4.9	23.9*

*Kitchen staff differ significantly from service staff at p<0.001 by Mann–Whitney U test; †summed PAHs: sum of pyrene, benzo(k)fluroranhene, benzo(a)pyrene, benzo(ghi)perylene and dibenzo(a,e)pyrene.

Table 3 lists urinary MDA and 1-OHP levels together with smoking status and job title. All kitchen staff had urinary MDA levels and 1-OHP levels significantly higher than those of service staff. For the non-smoking kitchen staff, mean MDA and 1-OHP levels were 344.2 (SD 243.7) and 6.0 (SD 8.0) μmol/mol creatinine, respectively, and thus were significantly higher than for non-smoking service staff, which were 244.2 (SD 164.4) and 2.4 (SD 4.3) μmol/mol creatinine, respectively. Urinary MDA levels in smoking kitchen staff remained significantly higher than those in service staff. However, urinary 1-OHP levels in smokers did not differ significantly between kitchen and service staff.

**Table 3:** Comparisons of urinary malondialdehyde and 1-hydroxypyrene levels between kitchen (n = 171) and service staff (n = 117)

Marker	Smoking status	Service staff	Kitchen staff
MDA	Smoker (n = 134)	293.3 (213.7) (n = 56)	398.0 (380.0) (n = 78)*
	Non-smoker (n = 154)	244.2 (164.4) (n = 61)	344.2 (243.7) (n = 93)†
	All (n = 288)	267.2 (189.9) (n = 117)	369.0 (314.0) (n = 171)‡
1-OHP	Smoker (n = 134)	6.9 (8.8) (n = 56)	6.5 (8.4) (n = 78)
	Non-smoker (n = 154)	2.4 (4.3) (n = 61)*	6.0 (8.0) (n = 93)†
	All (n = 288)	4.4 (7.0) (n = 117)*	6.2 (8.2) (n = 171)*

*Kitchen staff differ significantly from service staff at p<0.05 by Student’s t test; †kitchen staff differ significantly from service staff at p<0.01 by Student’s t test; ‡kitchen staff differ significantly from service staff at p<0.001 by Student’s t test.

Values are mean (SD) (μmol/mol creatinine). 1-OHP, 1-hydroxypyrene; MDA, malondialdehyde.

Table 4 lists the results of multiple linear regression analysis for urinary 1-OHP and MDA in non-smoking restaurant workers. Kitchen work was found to be a significant and positive predictor of urinary 1-OHP levels after adjusting for other covariates. Kitchen staff urinary 1-OHP levels were increased compared to those of service staff. Work years, work days per week and work hours per day were all marginally significant predictors of urinary 1-OHP (0.05<p<0.1). Cooking at home, second-hand smoke, age and BMI were not significant predictors of urinary 1-OHP levels for restaurant workers. Table 4 also lists predictors of urinary MDA levels for restaurant workers based on multiple linear regression analyses. This study found urinary excretion of 1-OHP and work hours per day to be the main predictors of MDA in urine for all workers after controlling for other covariates. An increase in urinary 1-OHP was associated with an increase in urinary MDA (p<0.001) and an increase in work hours per day was also associated with an increase in urinary MDA (p<0.05). Notably, work in kitchens was not a significant predictor of urinary MDA and work hours per day did not significantly predict urinary 1-OHP.

**Table 4:** Multiple linear regression analysis: predictors of urinary 1-hydroxypyrene and malondialdehyde levels in 154 non-smoking restaurant workers

Predictors	Log_10_ 1-OHP(μmol/mol creatinine)Regression coefficient (95% CI)	Log_10 _MDA(μmol/mol creatinine)Regression coefficient (95% CI)
Work in kitchens (kitchen vs service staff)	0.494 (0.160 to 0.827)*	0.090 (−0.022 to 0.203)
Cooking at home (yes vs no)	0.34 (−0.238 to 0.507)	0.034 (−0.087 to 0.156)
Second-hand smoke exposure (yes vs no)	0.185 (−0.128 to 0.497)	0.082 (−0.020 to 0.185)
Work years (years)	0.016 (−0.002 to 0.034)	0.003 (−0.001 to 0.009)
Work days per week (days)	0.484 (−0.003 to 0.971)	0.005 (−0.157 to 0.166)
Work hours per day (hours)	0.068 (−0.003 to 0.138)	0.025 (0.001 to 0.048)*
Age (years)	−0.002 (−0.019 to 0.015)	<0.001 (−0.006 to 0.005)
BMI (kg/m^2^)	−0.007 (−0.053 to 0.039)	<0.001 (−0.016 to 0.014)
Log_10_ 1-OHP (μmol/mol creatinine)	–	0.065 (0.032 to 0.111)**

1-OHP, 1-hydroxypyrene; MDA, malondialdehyde.

*p<0.05; **p<0.001.

## DISCUSSION

The high particulate PAH levels in Chinese restaurants demonstrated that kitchen staff have high exposure and service staff low exposure to COF. Since restaurant workers did not wear respiratory protection devices while working, they were at high risk of PAH exposure from COFs.

**Main messages**

- Urinary 1-hydroxypyrene and malondialdehyde levels in Chinese restaurant workers reflect occupational exposure to polycyclic aromatic hydrocarbons from cooking oil fumes and oxidative stress.
- Kitchen staff experience high exposure to cooking oil fumes in Chinese restaurants.

**Policy implications**

- Exposure to cooking oil fumes should be monitored in Chinese restaurants.
- Further surveys should be undertaken in Chinese restaurants to examine the relationship between exposure to cooking oil fumes and disease.
- Work place exposure to cooking oil fumes should be minimised.

This study used urinary MDA as a biomarker of oxidative stress, and urinary 1-OHP and work experience as indicators of COF exposure to study the impact of COFs on oxidative stress in Chinese restaurant workers. The linear multiple regression models were used to analyse non-smokers only owing to the strong effects of cigarette smoking on PAH uptake. Cigarette smoking influences urinary 1-OHP levels in restaurant workers, a finding which is consistent with a previous study on urinary 1-OHP in asphalt paving workers.^25^ Cooking at home is not a significant predictor of urinary 1-OHP levels. Cooking methods frequently used in restaurant kitchens included deep frying, stir frying and grilling, all of which generate significant quantities of COFs.^8^ ^26^ In contrast, steaming and stewing (neither of which generate significant amounts of COFs) were the main cooking methods used at home by restaurant workers, perhaps because they realise the hazards of COFs as a result of having attended government training programs. On the other hand, home cooking for a few minutes versus cooking in a restaurant for hours may be another reason why home cooking is not a significant predictor for COF exposure. The likelihood of second-hand smoke exposure at work is small because all 19 surveyed restaurants were non-smoking areas, including both the kitchen and dining areas. Thus, restaurant workers who smoked usually did so outdoors or in designated smoking areas. Second-hand smoke exposure did not significantly influence urinary 1-OHP levels because our definition tended to overestimate workers’ second-hand smoke exposure. This finding agrees with a previous study on urinary 1-OHP in police officers.^27^

This study also found, using MDA as an indicator, that oxidative stress in restaurant workers is positively associated with urinary 1-OHP levels and work hours per day. Thus, urinary 1-OHP was a good exposure biomarker for urinary MDA or oxidative stress.

One limitation of this study was the lack of PAH exposure data for non-occupational settings, such as from vehicle traffic emissions. However, each day the restaurant workers spent more than 10 h in the restaurants, including work and rest periods, compared to less than 1 h in traffic. Therefore, the contribution of traffic sources to the PAH exposure of restaurant workers is believed to be small. Another limitation of this study is the lack of information regarding individual susceptibility to COF exposure in our subjects, which we believe could bias our work. Regardless of this limitation, this study concluded that 1-OHP was a good predictor of urinary MDA in male workers in Chinese restaurants.

## CONCLUSIONS

The study findings indicate that urinary 1-OHP and MDA reflect occupational exposure to PAHs from COFs and oxidative stress in male workers in Chinese restaurants.
